# Adrenocorticotropic Hormone-Dependent Cushing's Syndrome Complicated With Gastric Ulcer Perforation in a 30-Year-Old Saudi Female: A Case Report and a Review of the Literature

**DOI:** 10.7759/cureus.48089

**Published:** 2023-11-01

**Authors:** Majed M Aladwani, Mishari T Alrubaiaan, Yazeed H Alrayani, Tareq N Alabdali

**Affiliations:** 1 Endocrinology, King Abdulaziz Medical City Riyadh, Riyadh, SAU; 2 College of Medicine, King Saud Bin Abdulaziz University for Health Sciences, Riyadh, SAU

**Keywords:** endocrine, hypercortisolism, gastro-intestinal perforation, cushing's syndrome, acth-dependent cushing syndrome

## Abstract

Gastrointestinal perforation is a well-addressed complication of exogenous hypercortisolism; however, patients with endogenous Cushing's syndrome (CS) do not usually experience this condition in clinical practice. The literature on this subject is limited and consists solely of clinical case reports/series with only 23 instances of gastrointestinal perforation occurring in individuals with endogenous Cushing's syndrome. This is mainly attributed to the rarity of Cushing's syndrome itself and the low chance of occurrence of such complications. We report a case of a recently diagnosed adrenocorticotropic hormone (ACTH)-dependent Cushing's syndrome in a 30-years-old female who presented initially with a three-month history of progressive weight gain, generalized weakness, acne, menstrual irregularity, and severe hypokalemia, and then developed a gastric ulcer perforation only one month after her ACTH-dependent Cushing's syndrome diagnosis and was managed through emergent surgery.

## Introduction

A disorder of the endocrine system characterized by excessive cortisol production, known as Cushing's syndrome, rarely occurs. The main causes are pituitary tumors, ectopic adrenocorticotropic hormone (ACTH)-secreting tumors, or adrenal tumors that secrete cortisol independently [1]. Patients initially present with a wide range of symptoms, including weight gain, proximal myopathy, skin thinning, and abdominal striae [1]. Additionally, several metabolic disorders, such as diabetes mellitus, hypertension, and dyslipidemia, can occur, especially when the diagnosis is not established at an early stage [2]. There is a possibility of gastrointestinal complications among patients receiving exogenous glucocorticoids. However, there is limited information on gastrointestinal complications associated with endogenous hypercortisolemia [3,4]. Thus far, only 23 instances have been published addressing the co-occurrence of gastrointestinal perforation with endogenous Cushing's syndrome [5-17]. To the best of our knowledge, this is the first case reporting gastric perforation in an ACTH-dependent Cushing's syndrome, while the vast majority reported diverticular, sigmoid, or duodenal perforation with Cushing's syndrome [5-17]. Herein, we describe the medical history, physical examination, and investigatory findings of a 30-year-old female with a recent diagnosis of ACTH-dependent Cushing's syndrome that was complicated by gastric ulcer perforation, necessitating an urgent exploratory laparotomy. The primary motivator of this case report was the rarity of the described condition, the atypical location of the perforation in such patient group, and the relatively young age of the patient.

## Case presentation

History and examination

A 30-year-old female with a history of mental retardation was admitted to our emergency department (ER) with progressive weakness and fatigue. Upon taking the history, she had been having menstrual irregularities, progressive weight gain, and generalized weakness, which was significant enough to limit her physical activity and hinder her movement for the past three months. Initial vital signs showed that the patient had a body temperature of 37°C, a pulse rate of 90 beats per minute, and a blood pressure of 130/80 mmHg. On physical examination, the patient had a moon face with supraclavicular fullness, dorsocervical fat pad, purple abdominal striae, facial signs of hirsutism, and acne all over the face, shoulders, chest, and back. 

Investigations

In the initial laboratory examination, hypokalemia of 2.1 mEq/L, hyperglycemia of 12.1 mmol/L, and metabolic alkalosis were detected (Table 1). The cortisol level after 1 mg dexamethasone suppression test was 2204 nmol/L (normal range 140-690), ACTH 123 pg/mL (normal range 7.2-63.3), DHEA-S 27.85 umol/L (normal range 2.6-13.9), And 24-hour urine cortisol level was 1560 mg/day (normal range 30-350) (Table 1). No suppression was observed in cortisol level with 8 mg dexamethasone suppression test. 

**Table 1:** Laboratory findings on initial presentation and on perforation day TSH - thyroid stimulating hormone; ACTH - adrenocorticotropic hormone

Parameter	Initial presentation	Perforation presentation	Refrence range
Na+	143 mEq/L	139 mmol/L	135-147 mEq/L
Cl-	85 mEq/L	105 mmol/L	98-108 mEq/L
K+	2.1 mEq/L	2.8 mmol/L	3.5-5.0 mEq/L
Mg2+	0.79 mmol/L	0.77 mmol/L	0.85-1.110 mmol/L
PO3-	0.88 mmol/L	1.23 mmol/L	0.97-1.46 mmol/L
PH	7.54	7.36	7.35-7.45
PCO2	67.5 mmHg	42.7 mmHg	35-45 mmHg
PO2	27.7 mmHg	62.2 mmHg	75-100 mmHg
HCO3	49.8 mEq/L	23.6 mEq/L	22-26 mEq/L
Random blood glucose	12.1 mmol/L	24.1 mmol/L	<5.5 mmol/L
Hemoglobin	13.5 g/dL	14.9 g/dL	13.7-16.8 g/dL
White blood cells	9,720 /uL	11,100 /uL	3,300-8,600 /uL
Lymphocyte	0.48%	0.33%	-
Neutrophil	8.55%	9.66%	-
Eosinophil	0.0%	0.0%	-
TSH	0.55 mIU/L	Was not ordered	0.4-4.0 mIU/L
Cortisol	2204 nmol/L	4842 nmol/L	140-690 nmol/L
ACTH	123 pg/mL	Was not ordered	7.2-63.3 pg/mL

A series of CT scans for the neck, chest, abdomen, and pelvis was performed and failed to localize any tumors acting as an ectopic source. A pituitary MRI was performed, and no adenoma was found. To complete the diagnostic workup, we decided to do an inferior petrosal sinus sampling (IPSS) and PET scan with Gallium 68; however, the patient's family refused and requested discharge and outpatient follow-ups. These results, together with the biochemical and clinical findings, supported the diagnostic hypothesis of ACTH-dependent Cushing's syndrome. 

Treatment/management

When addressing the issue of hypokalemia that the patient presented with initially, it was found to be resistant and difficult to correct. The patient was put on spironolactone 50 mg BID, and potassium chloride 20 mEq q8h, and her potassium level barely reached 3.5 mmol/L after several days. In addition, her magnesium level was corrected with magnesium oxide 800 mg every six hours. Her blood glucose level was controlled with insulin glargine 6 units daily and Novorapid as per the sliding scale. The patient was discharged on spironolactone tablets 50 mg BID (oral), potassium chloride 20 mEq q8h, cholecalciferol, calcium carbonate, insulin glargine 6 units daily, and Novorapid 4 units TID before meals.

Follow-up and outcomes

Seven days after discharge, she presented to the ER complaining of a new onset of abdominal pain, constipation, and reduced urine output. Her Glasgow Coma Scale (GCS) was 15, her blood pressure measurement was 146/90 mmHg, her pulse rate was 66 beats per minute, her respiratory rate was 21 breaths per minute, and her temperature was 36.7°C. Upon physical examination, the patient had distended non-tender abdomen without any other significant findings. Blood work was done, including renal functions, and all parameters, including potassium, were within normal limits. A chest X-ray was also performed and revealed no evidence of pneumoperitoneum. The patient was clinically stable after managing her abdominal pain with acetaminophen injection and administering fleet enema for constipation. After instructions on when to come again to the ER were given, the patient was discharged home on lactulose and paracetamol, and a close outpatient follow-up appointment was scheduled. 

**Figure 1:**
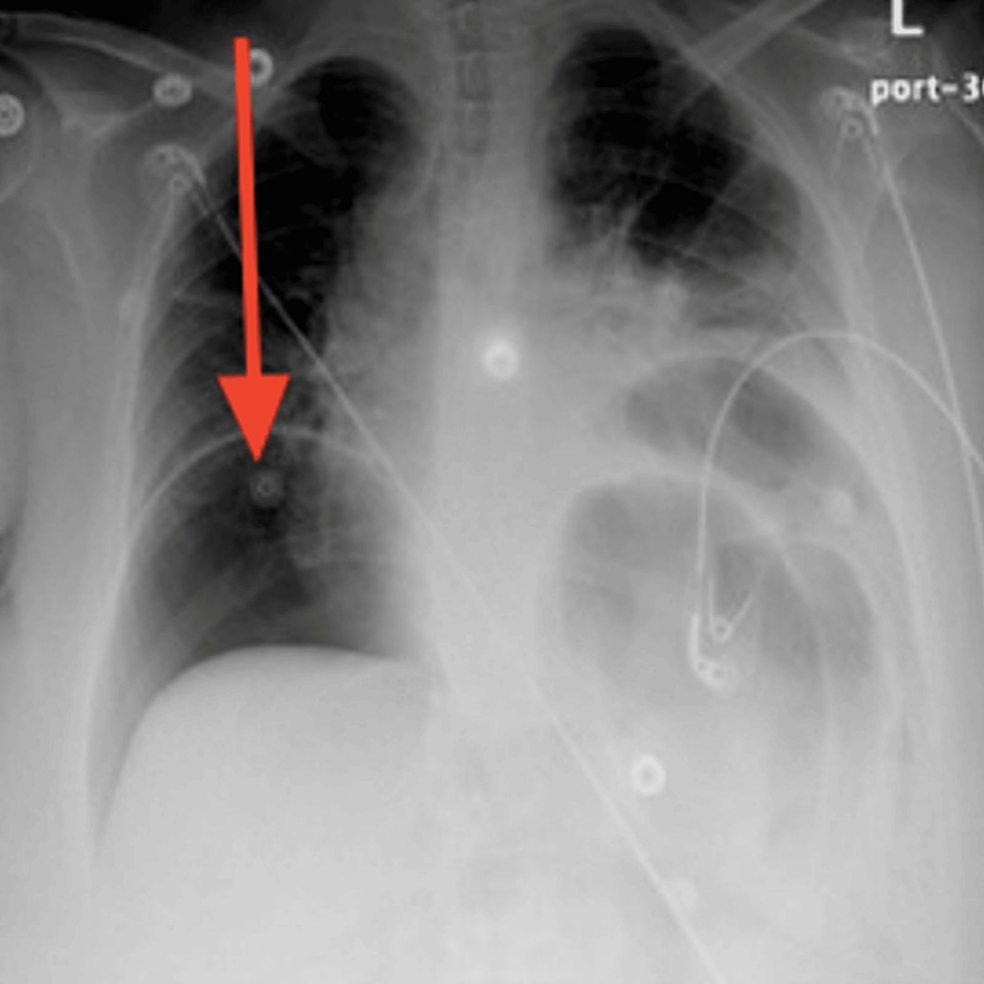
Posteroanterior chest X-ray at the time of gastric perforation displaying severe air under the diaphragm with bilateral obstruction indicating massive pneumoperitoneum (red arrow)

Five days after the ER visit, the patient presented again to the ER. She was still complaining of severe non-resolving abdominal pain, constipation, and reduced urine output. Upon physical examination in the ER, the patient was found to have developed a new onset of lower limb edema, abdominal rebound tenderness, and abdominal rigidity and guarding. She was hypotensive with a blood pressure of 91/46 mmHg, pulse rate of 80 beats per minute, respiratory rate of 16 breaths per minute, temperature of 38.2 °C, and SpO2 of 96%. The only significant laboratory finding was her potassium level dropping low to 2.8 mEq/L (Table 1). An X-ray of the chest was requested and showed a large pneumoperitoneum (Figure 1).

**Figure 2:**
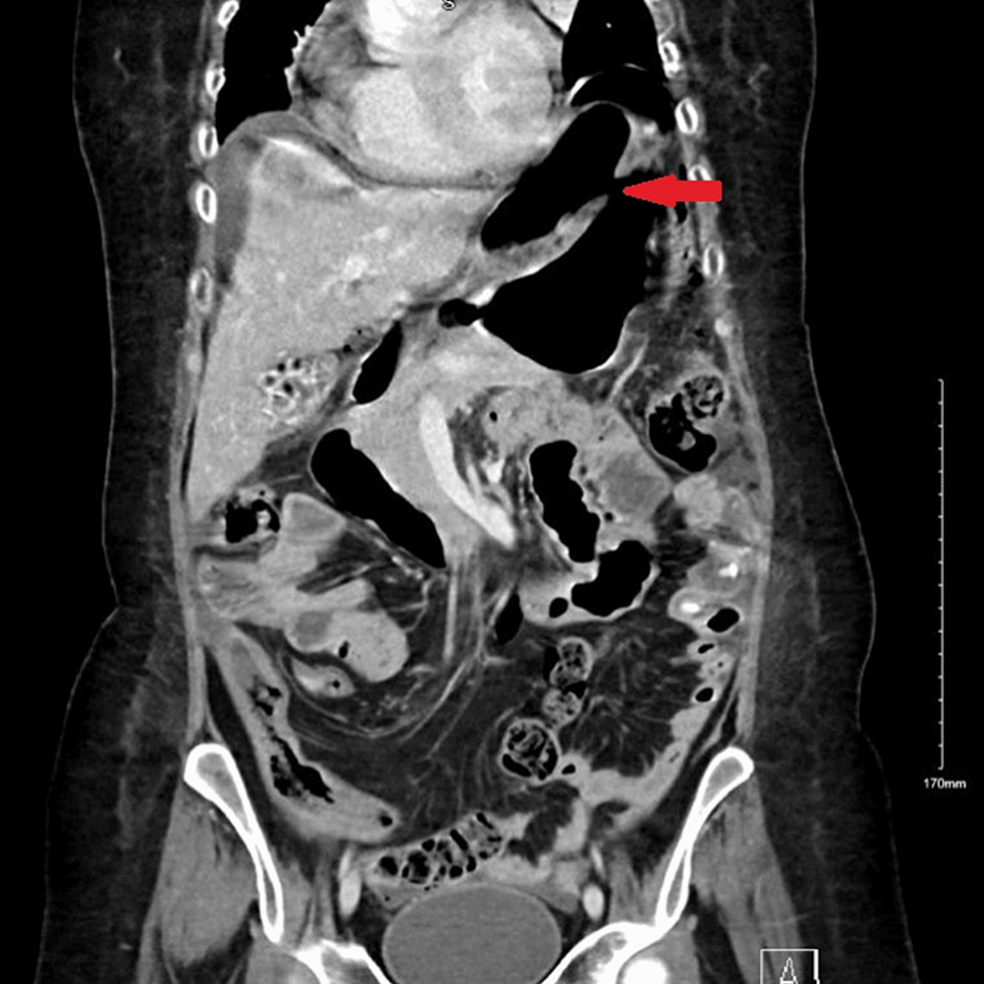
Coronal-section CT image of abdomen and pelvis at the time of gastric perforation showing features of gastric perforation likely related to the underlying perforated peptic ulcer with 0.8 cm defect at the distal greater curvature

**Figure 3:**
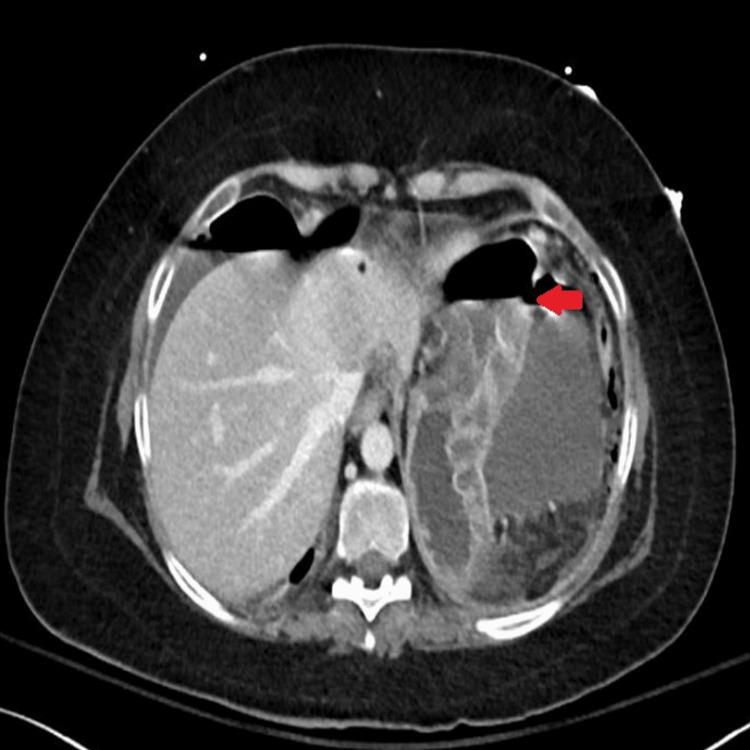
Horizontal-section CT image showing features of gastric perforation likely related to the underlying perforated peptic ulcer with 0.8 cm defect at the distal greater curvature

Abdominal CT was also urgently performed and confirmed the presence of gastric perforation likely related to an underlying perforated peptic ulcer with 0.8 cm defect at the distal greater curvature (Figures 2, 3).

The patient underwent an emergent gastric wedge resection for gastric perforation, and the pathology reported evidence of gastric ulcer with no evidence of malignancy. Furthermore, *Helicobacter pylori* test was performed on the sample, and it came back positive. The patient tolerated the surgery very well, and postoperative recovery was without any complications.

Later, the patient was prescribed metyrapone 250 mg Q4h, which was then increased to 500 mg Q4h four days after surgery, and her cortisol level significantly dropped to 634nmol/L. During that time, a gastrin level test was also performed to exclude the presence of gastrinomas, and the level was 45 pg/ml (normal range 13-115). 

## Discussion

A small percentage of the population suffers from Cushing's syndrome, which is an endocrine disorder characterized by an endogenous overproduction of glucocorticoids, resulting in hypercortisolemia [1]. It is estimated to affect 0.7 to 2.4 people per million annually [1]. Hypercortisolemia alters psychologic, metabolic, and cardiovascular functions, resulting in increased mortality and morbidity rates, particularly if the diagnosis is delayed and long-term exposure to high cortisol levels occurs [2]. Women are more likely to suffer from this condition than men, and people in their 40s to 60s are most vulnerable to it [1]. Patients initially present with a wide range of symptoms, including weight gain, proximal myopathy, skin thinning, and abdominal striae [1]. Additionally, several metabolic disorders, such as diabetes mellitus, hypertension, and dyslipidemia, can occur [1]. Due to the rarity of this condition, there is often a significant delay in diagnosis and treatment, which could eventually lead to complications from prolonged hypercortisolism. 

From another standpoint, in a systematic review, the incidence of peptic ulcer perforation ranges from 3.8 to 14 per 100,000 individuals in the general population [18]. In under-developed countries, patients are typically young, tobacco-using males [19]. However, patients in industrialized countries are typically older with multiple co-morbidities and are on long-term non-steroidal anti-inflammatory drugs (NSAIDs) or steroid use [19]. Patients may present with an abrupt onset of abdominal discomfort, abdominal rigidity, and tachycardia in the early stages of a perforated peptic ulcer [19]. Later, abdominal distention, pyrexia, hypotension, fever, and vomiting can occur [19]. Furthermore, when the diagnosis is made early, a perforated ulcer often has a good prognosis. However, the risk of adverse events increases if there is a delay in the diagnosis [20]. Therefore, making an early detection through different imaging modalities is crucial [20]. A history of peptic ulcer disease, NSAIDs, physiological stress, smoking, corticosteroids, and *Helicobacter pylori* are some of the well-established risk factors for a perforated peptic ulcer [20]. 

The prevalence of *Helicobacter pylori* among Saudi patients is high; in one study, the overall prevalence was 46.5% in patients with dyspepsia using gastric biopsy [21]. Several studies have explored the relationship between *Helicobacter pylori* and gastrointestinal perforation, but the results have been mixed. Some studies have suggested a higher prevalence of *Helicobacter pylori* infection among individuals with gastrointestinal perforation compared to those without, indicating a potential association. However, other studies have found no significant difference in the prevalence of *Helicobacter pylori* infection between perforated and non-perforated gastrointestinal ulcer cases [22]. Furthermore, they suggested that the presence of other risk factors like the use of NSAIDs, smoking, and alcohol may interact with *Helicobacter pylori* infection and contribute to the development of complications such as gastrointestinal perforation [22]. However, in our case, the patient did not have any established risk factors for gastric perforation, such as NSAIDs, smoking, or alcohol. Therefore, considering the low incidence of gastrointestinal perforation and high prevalence of *Helicobacter pylori*, the conflicting data regarding the association between *Helicobacter pylori* and gastrointestinal perforation, and the lack of established risk factors for gastrointestinal perforation in our patient, we suggest that prolonged excess glucocorticoids from Cushing's syndrome may have contributed to the gastric perforation either independently or synergistically with *Helicobacter pylori* since hypercortisolism can lead to a weakened gastrointestinal wall integrity due to decreased collagen turnover and disruption of mucosal protection by prostacyclin [15]. In addition, because of hypercortisolism, perforation may not be contained or healed initially due to the immunosuppressive effects of hypercortisolism, whether endogenous or exogenous [15]. Additionally, high levels of cortisol may delay the diagnosis and treatment since it may mask the symptoms of the perforation [14]. Moreover, our patient was treated for severe hypokalemia with potassium supplementation for an extended period of time. Previous studies have linked potassium chloride supplementation to gastrointestinal ulceration and perforation, making this a possible additive cause of our patient's condition [23,24].

A limited number of studies have addressed gastrointestinal perforations associated with endogenous hypercortisolemia [5-17]. The correlation between Cushing's syndrome and gastrointestinal perforation is highlighted in our study and in the case reports that have been previously published (Table 2). Similar to our case, a female predominance was seen in gastrointestinal perforation among the reported cases of Cushing's syndrome [6,7,12,13,15,16]. Additionally, the average age at which gastrointestinal perforation occurred in patients with endogenous hypercortisolism ranged from 45 to 80, which is a noticeably higher age range than the case we are presenting here (aged 30) [6-10,12]. Furthermore, unlike our case, in which gastrointestinal perforation occurred four months after the onset of Cushing's symptoms, Intestinal perforation occurs approximately 9.8 months after Cushing's symptoms first appear [15]. Furthermore, in our patient, gastric perforation occurred while she was hypercortisolemic and not in a remission state. Hence, in association with *Helicobacter pylori* infection, severe hypercortisolemia could have been a secondary contributing factor to gastric perforation. The complications of gastric ulceration, specifically with endogenous Cushing's syndrome, have been addressed in two case reports [25,26]. It must be noted, however, that neither case is similar to ours. A case of gastric perforation was reported by Kubicka et al. in a patient who had a confirmed diagnosis of gastrinoma, and the patient was diagnosed with ectopic Cushing's syndrome seven months after gastric perforation [25]. Therefore, since ectopic Cushing's syndrome was diagnosed seven months after the perforation, it is more likely that the gastrinoma contributed to this complication. In contrast, our patient's serum gastrin level was within the normal range, ruling out gastrinoma. Further, Hoshino et al. reported a case of gastrointestinal bleeding in a 39-year-old man with a confirmed diagnosis of Cushing's disease secondary to pituitary adenoma [26]. He was found to have gastric ulceration and bleeding along with *Helicobacter pylori* infection and elevated cortisol levels [26]. In spite of the patient not developing a gastric perforation, it was suggested by the author that hypercortisolism might be a contributing factor for gastric ulcer complications by slowing down the ulcer healing process [26]

**Table 2:** Current case and previous reported 23 cases of patients with Cushing's syndrome and gastrointestinal perforation UFC - urinary free cortisol; PC - plasma cortisol; ACTH - adrenocorticotropic hormone

Reference	Year of publication	Age, gender	Highest cortisol level plasma cortisol (PC, nmol/L) / UFC (nmol/L)	Cause of Cushing's syndrome	Time from onset of Cushing's symptoms to perforation (months)	Reported site of gastrointestinal perforation
Current	2023	30, Female	PC 4842	ACTH-dependant	4	Gastric perforation
Ishinoda et al. [17]	2023	24, Male	PC 1647	Cushing's disease	12	Sigmoid colon perforation
Wijewickrama et al. [16]	2021	32, Female	PC 1147	Pituitary microadenoma	1	Diverticular perforation
Shahidi et al. [15]	2019	72, Female	UFC 5296	Pancreatic neuroendocrine tumor	12	Diverticular perforation
Shahidi et al. [15]	2019	61, Female	PC 1925	Metastatic medullary carcinoma of thyroid	12	Sigmoid colon and diverticular perforation
Shahidi et al. [15]	2019	68, Female	UFC 410	Cushing's disease	12	Sigmoid colon perforation
Shahidi et al. [15]	2019	71, Female	UFC 1533	Cushing's disease	4	Diverticular perforation
Shahidi et al. [15]	2019	54, Male	UFC 374	Cushing's disease	3	Sigmoid colon perforation
Shahidi et al. [15]	2019	52, Female	UFC 885	Cushing's disease	16	Diverticular perforation
Sater et al. [14]	2018	80, Female	UFC 5601	Lung carcinoid	36	Diverticular perforation
Sater et al. [14]	2018	60, Female	UFC 72726	Metastatic islet cell carcinoma	36	Diverticular perforation
Sater et al. [14]	2018	31, Male	UFC 1297	Cushing's disease	20	Diverticular perforation
Sater et al. [14]	2018	52, Female	UFC 2371	Lung carcinoid	4	Diverticular perforation
Sater et al. [14]	2018	67, Male	UFC 3836	Ectopic ACTH	10	Diverticular perforation
Sater et al. [14]	2018	51, Male	UFC 13552	Metastatic thymic carcinoma	4	Diverticular perforation
Kaya et al. [9]	2016	70, Male	PC 1432	Small cell lung cancer	1	Diverticular perforation
Dacruz et al. [12]	2016	60, Female	UFC 4481	Metastatic parotid tumor	5	Sigmoid colon and diverticular perforation
Matheny et al. [10]	2016	67, Male	UFC 11119	Metastatic medullary carcinoma of thyroid	4	Diverticular perforation
Flynn et al. [13]	2016	63, Female	UFC 12465	Pheochromocytoma	1	Perforation at the splenic flexure
Balestrieri et al. [11]	2016	75, Male	PC 2272	Neuroendocrine tumor	1	Intestinal perforation
Hara et al, [8]	2013	79, Male	PC 1230	Cushing's disease	6	Diverticular perforation
De Havenon et al. [7]	2011	71, Female	PC 2593	Cushing's disease	9	Diverticular perforation
Lutgers et al. [6]	2010	55, Female	UFC 10152	Right pheochromocytoma	1	Sigmoid colon and diverticular perforation
Drake et al. [5]	1998	35, Male	PC 1442	Islet cell tumor	4	Duodenal perforation and rupture of pancreatic pseudocyst

## Conclusions

A high blood cortisol level can be associated with various clinical manifestations and diverse sets of complications. This case report sheds light on one of the less common complications of hypercortisolism in patients with Cushing's syndrome, which is gastrointestinal perforation. Our report further supports the published evidence that gastrointestinal perforation is a rare but potentially fatal complication among patients with Cushing's syndrome. Moreover, it highlights the possibility of developing gastric perforations in this patient group, even at younger ages than expected. This should elicit a high clinical suspicion and demand prompt investigation of Cushing's syndrome patients in a hypercortisolism state presenting with modest gastrointestinal symptoms.
